# Massive odontoameloblastoma arising in the maxilla: a case report

**DOI:** 10.1186/s13256-015-0743-0

**Published:** 2015-12-08

**Authors:** Masanori Kudoh, Hiroyuki Harada, Yuriko Sato, Ken Omura, Yoshimasa Ishii

**Affiliations:** 1 Division of Oral and Maxillofacial Surgery, Ebina General Hospital, 1320, Kawaraguchi, Ebina, Kanagawa, 243–0433 Japan; 2 Oral and Maxillofacial Surgery, Department of Oral Restitution, Division of Oral Health Sciences, Graduate School, Tokyo Medical and Dental University, 1–5–45 Yushima, Bunkyo-ku, Tokyo, 113–8549 Japan; 3 Division of Oral and Maxillofacial Surgery, General Tokyo Hospital, 3–15–2 Ekota, Nakano-ku, Tokyo, 165–0022 Japan

**Keywords:** Maxillary sinus, Mixed odontogenic tumor, Odontoameloblastoma

## Abstract

**Introduction:**

Odontoameloblastoma is an extremely rare mixed odontogenic tumor with both epithelial and mesenchymal components. The term *odontoameloblastoma* first appeared in the 1971 World Health Organization classification (Pindborg JJ., et al.) and is defined as “a neoplasm that includes odontogenic ectomesenchyme in addition to odontogenic epithelium that resembles an ameloblastoma in both structures and behavior.” Because of the aggressive nature and risk of recurrence of the tumor, complete resection is essential. In this report, we describe an extremely rare case of a patient with massive odontoameloblastoma arising in the maxilla and occupying maxillary sinus.

**Case presentation:**

In 2013, an 11-year-old Japanese boy was referred to our department for a painless and large mass of the right maxillary region. A panoramic X-ray showed a unilocular cystic lesion in the right maxilla containing a calcified mass in the lesion associated with an impacted tooth. Computed tomography showed a cystic lesion that included calcified structures and measured 3.6×3.1×2.7 cm. In 2013, the patient underwent tumor extirpation combined with impacted tooth extraction. The histopathological diagnosis was an odontoameloblastoma. No recurrence was noted 27 months after the operation.

**Conclusions:**

The patient has undergone postoperative occlusal guidance and functional orthodontic treatment, and his postoperative condition is excellent. However, postoperative recurrence or malignant transformation can occur in cases of odontoameloblastoma, and close long-term follow-up will be continued for our patient.

## Introduction

Odontoameloblastoma (OA) is an extremely rare mixed odontogenic tumor that is defined in the current World Health Organization tumor classification system as a tumor that includes odontogenic ectomesenchyme and odontogenic epithelium and resembles an ameloblastoma in both structure and behavior [1]. Generally, the clinical presentation of OA resembles an odontoma; thus, a definitive diagnosis is based on histologic analysis following excision and curettage [2]. OA and complex odontoma belong to a group of odontogenic tumors that consist of odontogenic epithelium and odontogenic ectomesenchyme with or without dental hard tissue formation (so-called mixed odontogenic tumors) [3, 4]. However, differential diagnosis of OA is difficult compared with ameloblastic fibroodontoma or a developing complex odontoma [5].

OA is usually found in young patients and has no significant gender predilection [6, 7]. Clinically, the two main complaints are swelling and failure of tooth eruption. Radiological examination usually reveals a multilocular radiolucency with a well-defined boundary and often shows radiopaque areas resembling mature dental tissue [8]. If an unerupted tooth is present, the tumor is usually situated coronally to the crown of this tooth [6, 8]. We report an extremely rare case of a patient with massive OA arising in the maxilla and occupying the maxillary sinus.

## Case presentation

**Fig. 1:**
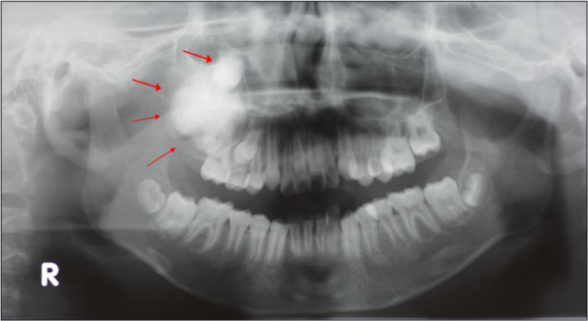
An unilocular cystic lesion in the right maxilla containing calcified mass in the lesion associated with impacted tooth (arrow)

**Fig. 2:**
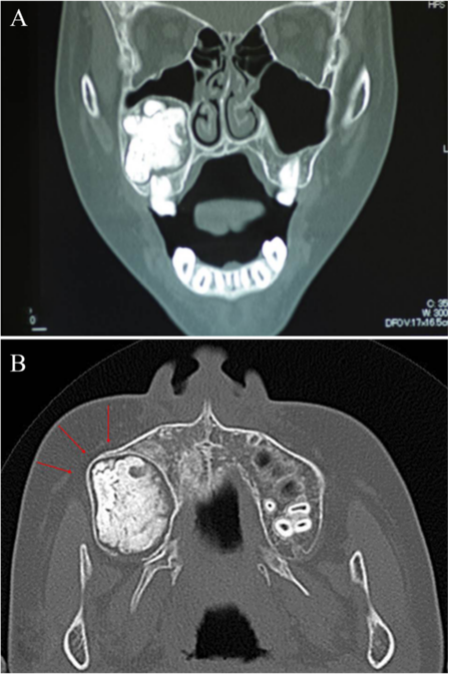
Computed tomographic images. **a** Axial view computed tomographic image showing a cystic lesion of size 3.6×3.1×2.7 cm that included calcified structures. **b** Horizontal view computed tomographic scan showing right maxillary bone expansion (*arrows*)

In 2013, an 11-year-old Japanese boy was referred to our department for painless bone expansion in the right maxillary alveolus, delayed eruption of the permanent second molar teeth, and altered occlusion. He had no significant medical or family history. A panoramic X-ray showed a unilocular cystic lesion in the right maxilla containing a calcified large mass associated with an impacted tooth (Fig. 1). Computed tomography showed a cystic lesion of size 3.6×3.1×2.7 cm that included calcified structures (Fig. 2a). A horizontal view showed right maxillary bone expansion (Fig. 2b).

In 2013, the patient underwent tumor extirpation combined with impacted tooth extraction. The incisional line was started from the labial mucosa in the right maxillary central and lateral incisor area. It extended to the gingiva in the right maxillary lateral incisor and canine area by arch-like incision, and to the gingiva of the right maxillary second molar by crestal incision with distal releasing incision. The goal of this procedure was to make a mucoperiosteal flap from the lower border of the pyriform aperture, vertically to the infraorbital foramen and surrounding areas, and horizontally to the lower border of the zygomatic bone. The infraorbital neurovascular bundles were preserved. Upon opening of the sinus from a thin plate of bone in the canine fossa and surrounding areas in the anterior wall of the right maxilla (the same level as the lower border of the pyriform aperture), bone-like hard tissues that were strongly adhered to the maxilla were found. When these tissues were separated from the surrounding areas and removed using a fissure bar, a solid, bone-like, hard odontogenic tumor (similar to a complex odontoma) was found to have occupied almost the whole sinus. The mass was too large to remove from the opening.

**Fig. 3:**
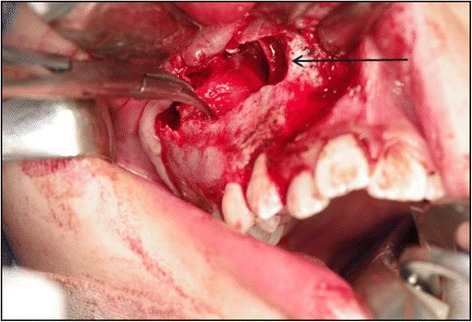
Intraoperative view showing extirpation of the tumor arising in the maxilla (arrow)

**Fig. 4:**
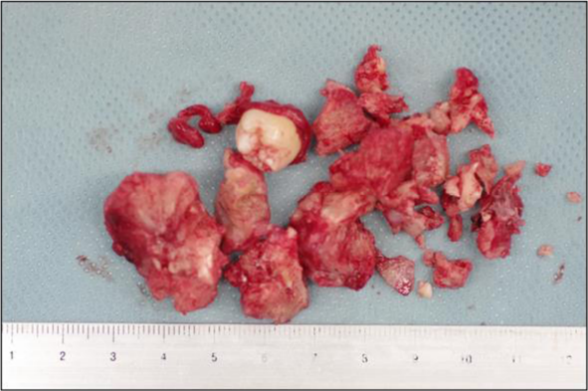
Surgical specimens

**Fig. 5:**
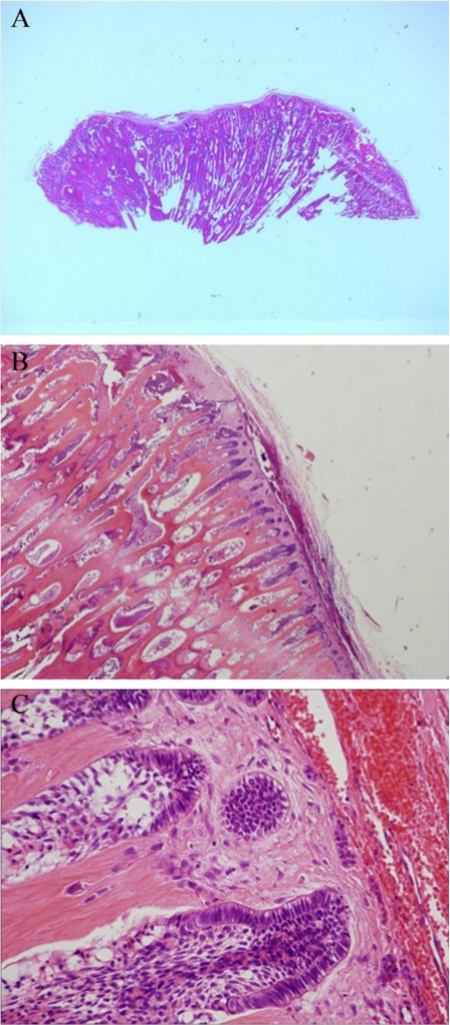
**a** Histopathological image (hematoxylin and eosin stain, original magnification ×100). A histological examination indicated the presence of a mixture of dentin and enamel with a radial structure. **b**, **c** Histopathological images (hematoxylin and eosin stain, original magnification ×200). A palisade arrangement of cylindrical cells was seen in the margin of the odontogenic epithelial-like cell structures, and stellate cells had proliferated in the alveolar structures

For complete removal of the mass including the tumor capsule, which was adhered to the surrounding bone, the tumor was divided into several pieces using a fissure bar (Fig. 3). There was an unerupted permanent tooth in the posterior part of the maxillary sinus anterior wall directly above the tumor resection site. This tooth appeared to have been pushed up by the tumor. It deviated from the dental arch and was included in the tumor body. This made preservation difficult, and the tooth was extracted (Fig. 4). A part of the root apex of the right maxillary first molar protruded through the tumor resection site, and conservative treatment was applied because of the patient’s age. There was no tooth germ of the unerupted second molar in the tumor resection cavity. There was access between the sinus and the tumor resection cavity, but there were no signs of maxillary sinusitis, which allowed use of conservative treatment. After hemostasis was confirmed, the incision was stitched closed. After the tumor extirpation, the wound was sutured with VICRYL absorbable stitches (Ethicon, Somerville, NJ, USA). Examination of the surgical specimen showed that the lesion consisted of various hard tissues, including a tooth-like structure (Fig. 4). A histological examination indicated the presence of a mixture of dentin and enamel with a radial structure (Fig. 5a). Fibrous tissues were observed between the hard tissues, which suggested mild mononuclear cell infiltration. Furthermore, the hard tissues were covered by fibrous tissues, and odontogenic epithelial-like cell structures were externally elongated from the inside to the outer boundary of the hard tissues. A palisade arrangement of cylindrical cells was seen in the margin of the odontogenic epithelial-like cell structures, and stellate cells had proliferated in the alveolar structures (Fig. 5b and c). Cellular atypism was unremarkable, and there were few Ki-67-positive cells. These features confirmed the diagnosis of an OA. The histopathological diagnosis was OA.

No recurrence was noted at 27 months after the operation. The patient has undergone postoperative occlusal guidance and functional orthodontic treatment, and his postoperative condition is excellent.

## Discussion

OA is an aggressive odontogenic tumor characterized by simultaneous occurrence of an ameloblastoma and a compound or complex odontoma in the same tumor mass [3, 9]. OA affects males and females equally and occurs in the maxilla and mandible, with the molar-premolar area being the most common site. Clinical symptoms include slow, progressive swelling of the alveolar plates, bone expansion, root resorption, dull pain, altered occlusion, tooth displacement, delayed eruption, and impacted teeth [6]. OA occurs between the ages of 2 and 50 years and at a mean age of 20.2 years [6]. However, as in our patient, most cases occur in children under 16 years of age.

The origin of an OA [10] has been proposed to be malignant transformation of the enamel epithelium after odontogenesis [11] or malignant transformation of the epithelium and mesenchyme of supernumerary teeth during embryogenesis [12]. However, a definitive theory has yet to be established [13]. Furthermore, the disease is often accompanied by an impacted tooth, but the causative link between the tumor and the impacted tooth is unclear. Thompson *et al*. [14] suggested that the tumor capsule may be derived from mesenchymal tissue, based on the property of this tissue to form dental hard tissue. The origin of our case was unclear, but between tumor and tumor capsule that similar to the dental follicle around the impacted tooth was connected, with irregular structures and dentinal hyperplasia was seen, we guessed tumor transformation was caused by interaction between the odontogenic epithelium which degenerated and mesenchymal tissue during the degenerated tooth process. This suggests that tumor formation may occur during the process of crown formation of the impacted tooth.

Ameloblastoma, complex odontoma, compound odontoma, and dentigerous cysts may be differentiated by imaging, but their diagnosis also requires histopathological examination. A definitive diagnosis is difficult to make on the basis of imaging and clinical findings alone; therefore, total excision is required. Masqueda-Taylor *et al*. [6] found recurrence in 3 (21.4 %) of 14 OA cases. On the basis of these data and findings that OA tends to occur at an earlier age than conventional ameloblastoma and because it has a similar potential to produce bone expansion, root resorption, and recurrence, it was suggested that OA should be treated similarly to ameloblastoma, with wide excision and close follow-up for at least 5 years [6]. Therefore, the first-choice treatment for OA is complete surgical extirpation of the tumor, including wide excision. We also suggest that treatment of OA requires close cooperation of oral and maxillofacial surgeons with orthodontic and pedodontic specialists, including primary care dentists. This multidisciplinary treatment is required because most cases of OA in pediatric patients are associated with displaced, unerupted permanent teeth.

Clinically, OA starts as a slow-growing, painless mass that expands the alveolus and vestibular cortex and prevents eruption of permanent teeth [9]. Our patient had expansion in the right maxillary alveolus, prevention of eruption of permanent teeth, altered occlusion, tooth displacement, delayed eruption, and impacted teeth. Most reported cases have had symptoms such as swelling or pain, but in our patient the tumor was asymptomatic and was detected during a routine examination. OA also tends to produce bone expansion in almost all cases, similarly to conventional ameloblastoma, whereas an odontoma seldom produces swelling of the affected region. Therefore, OA is often confused with compound or complex odontomas, as in our patient. Our patient has had no recurrence for 27 months postoperatively. However, the recurrence rate of OA is 21.4 % [6], and malignant transformation may occur [15]. Therefore, long-term follow-up will be continued for our patient.

## Conclusions

We report an extremely rare case of a patient with massive OA arising in the maxilla and occupying the maxillary sinus.

## Consent

Written informed consent was obtained from the patient’s legal guardian(s) for publication of this case report and any accompanying images. A copy of the written consent is available for review by the Editor-in-Chief of this journal.

## Abbreviation

OA: Odontoameloblastoma
